# A critical assessment of cross-species detection of gene duplicates using comparative genomic hybridization

**DOI:** 10.1186/1471-2164-11-304

**Published:** 2010-05-13

**Authors:** Heather E Machado, Suzy CP Renn

**Affiliations:** 1 Department of Biology, Reed College, Portland, OR 97202 USA

## Abstract

**Background:**

Comparison of genomic DNA among closely related strains or species is a powerful approach for identifying variation in evolutionary processes. One potent source of genomic variation is gene duplication, which is prevalent among individuals and species. Array comparative genomic hybridization (aCGH) has been successfully utilized to detect this variation among lineages. Here, beyond the demonstration that gene duplicates among species can be quantified with aCGH, we consider the effect of sequence divergence on the ability to detect gene duplicates.

**Results:**

Using the X chromosome genomic content difference between male *D. melanogaster *and female *D. yakuba *and *D. simulans*, we describe a decrease in the ability to accurately measure genomic content (copy number) for orthologs that are only 90% identical. We demonstrate that genome characteristics (e.g. chromatin environment and non-orthologous sequence similarity) can also affect the ability to accurately measure genomic content. We describe a normalization strategy and statistical criteria to be used for the identification of gene duplicates among any species group for which an array platform is available from a closely related species.

**Conclusions:**

Array CGH can be used to effectively identify gene duplication and genome content; however, certain biases are present due to sequence divergence and other genome characteristics resulting from the divergence between lineages. Highly conserved gene duplicates will be more readily recovered by aCGH. Duplicates that have been retained for a selective advantage due to directional selection acting on many loci in one or both gene copies are likely to be under-represented. The results of this study should inform the interpretation of both previously published and future work that employs this powerful technique.

## Background

It is well established that gene duplication and the subsequent evolution of duplicates is an important source of functional novelty [for review see [1]]. For example, gene duplications are known to be involved in adaptive evolution in response to diet [2-4], chemical challenge [5,6], and reproductive incompatibility [7,8]. Such adaptations can allow diversification into new niches, as has been suggested for cold adaptation [plants: [9], Antarctic ice fish: [10]] novel metabolic processes [C-4 photosynthesis: [11]]. Appreciation for the pervasive nature of gene duplication has been reinforced by genomic studies that identify dramatic variation in gene copy number between individuals and between species [[12], human: [13], mouse: [14,15], comparative mammals: [16], *Drosophila*: [17], *Arabidopsis*: [18]].

One genomic technique for identifying gene duplications among lineages is array-based comparative genomic hybridization (aCGH). This technique can identify duplicates that may be collapsed during shotgun sequence assembly [19]. Furthermore, unlike next-generation DNA sequencing technologies, this technique does not rely on a full genome assembly as a reference [[20], e.g. [21]]. In addition to the assessment of copy number variation within a species [e.g. [22]] and between closely related lineages [*Drosophila*: [23], *D. discoideum*: [24], experimental evolution in yeast: [25]], array CGH has been applied to the identification of chromosomal aberrations underlying cancer [for review see [26]] and genotyping of individuals within and between populations according to single nucleotide polymorphisms [e.g. *Arabidopsis*: [27], stickleback fish: [28]]. For each of these applications, when gDNA isolated from one sample is competitively hybridized against gDNA isolated from another sample, genomic regions that have been deleted (or are highly diverged) in the genome of the first sample will fail to hybridize to the array features resulting in a log ratio less than zero. Conversely, genomic regions that have been duplicated in the first sample will hybridize at a ratio of 2:1 (or greater), resulting in a log ratio greater than zero. When using aCGH to compare genome content between two different species, only one of these gDNA samples is isolated from the species for which the microarray was constructed, and the other gDNA sample is isolated from a heterologous species of interest. Through repetition with multiple heterologous species, a phylogenetic study can be performed that can address genome content in a broad range of related organisms. The aCGH technique has been used to reveal genomic regions likely involved in an organism's ability to inhabit a specific environment [*Chlamydia trachomatis *tissue specificity: [29], *Sinorhizobium meliloti *root symbiont: [30], *Clostridium difficile *host specificity: [31]], pathogenicity [[32], *Yersinia pesits*: [33], *Mycobacterium tuberculosis*: [34], *Vibrio cholerae*: [35]], genomic duplications and deletions associated with population divergence and speciation [*Anopheles gambiae*: [36,37]], and genomic regions that differentiate humans from other primate species [38,39].

Array-based genomic comparisons can also identify orthologs with high sequence divergence because an increased number of basepair differences between the platform species and the heterologous species will cause a detectable reduction in hybridization strength for the heterologous species [40,41]. We have shown a consistent linear relationship between hybridization ratio and sequence divergence. While 40% of the variation in hybridization ratio was accounted for by variation in sequence identity of the heterologous sample to the platform sample, other characteristics of the DNA sequence, such as GC content and alignment characteristics, also contributed to variation in hybridization ratio [40]. The extent to which reduced hybridization due to sequence divergence compromises the ability to accurately identify gene duplications has not been rigorously addressed, nor have the resultant biases in types of gene duplicates been identified. Here we quantify this effect by using genomic content as a model for gene duplication by specifically focusing on X-linked genes, such that these genes are "duplicated" in female individuals (XX) relative to male individuals (XY). Using three Drosophilid species for which complete genome assemblies are available [42], we survey thousands of orthologs over a range of sequence divergence. We quantify the ability to accurately detect increased genomic content of *D. simulans *and *D. yakuba *relative to *D. melanogaster *males using CGH on a *D. melanogaster *microarray. We find a decreased ability to identify genomic content with increased sequence divergence, suggesting that array CGH will be biased toward the identification of recent duplicates or otherwise conserved duplicates. We further discuss other potential confounding factors that may affect duplicate detection.

## Results

### High aCGH success for within-species duplicate detection

**Figure 1:**
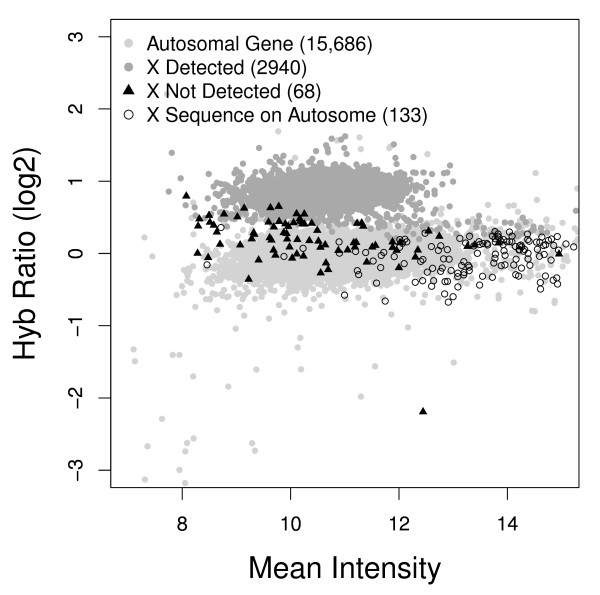
**Plot of *D. melanogaster *female vs. male genomic DNA**. X chromosome features identified as being in genomic excess in the female sample ("X Detected") and not identified as being in genomic excess ("X not Detected"), and the autosomal features identified as being in genomic excess that also share sequence similarity to an X chromosome sequence ("X Sequence on Autosome").

In the *D. melanogaster *male versus female analysis, 3146 of the 18849 analyzed features represented genes located on the X chromosome. Consistent with their X chromosome location, over 93% of these features were correctly identified as having greater genomic content in the female as measured by an increased log2 hybridization ratio that was statistically greater than zero (P < 0.1 FDR corrected)(Figure 1). Among the "false negatives" (X chromosome orthologs for which an excess genomic content was not identified by hybridization ratio), over half (138 of 206) are highly similar (E < 10^-14^) to one or more autosomal sequence. Furthermore, there was a small but significantly positive correlation (R = 0.181, P = 0.034) between the hybridization ratio and copy number ratio for false negatives.

### Successful detection of X chromosome orthologs in heterologous species

In order to assess the potential for aCGH to detect duplication events in a heterologous species relative to the array platform species, we tested for significantly female biased hybridization ratios using females from two additional species relative to *D. melanogaster *males. X chromosome orthologs were successfully found to have greater genomic content in both heterologous species, although at a reduced rate compared to the *D. melanogaster *within-species analysis. For *D. simulans*, approximately 37% of the analyzed X chromosome features were correctly identified, and for the more distantly related *D. yakuba *this true positive rate dropped to 26% (P < 0.1 FDR) (Table 1).

**Table 1:** Identification of genomic excess for array features that represent X chromosome genes.

Normalization	Test Species	True Positives (rate)	False Positives (rate)	False Negatives (rate)
1000 conserved genes	*D. melanogaster*	2940 (93%)	569 (16%)	206 (7%)
	*D. simulans*	1211 (37%)	274 (18%)	2021 (63%)
	*D. yakuba*	804 (26%)	662 (45%)	2243 (74%)
				
100 conserved genes	*D. melanogaster*	2921 (93%)	614 (17%)	225 (7%)
	*D. simulans*	944 (29%)	146 (13%)	2288 (71%)
	*D. yakuba*	522 (17%)	307 (37%)	2525 (83%)
				
All genes	*D. melanogaster*	2916 (93%)	372 (11%)	230 (7%)
	*D. simulans*	1685 (52%)	1079 (39%)	1547 (48%)
	*D. yakuba*	1698 (56%)	3404 (67%)	1349 (44%)

### Reduced false positive rate using conserved genes for normalization

The false positive rate (the percent of array features found to be in genomic excess that map to autosomes) was greater in the heterologous species than in *D. melanogaster *(*D. melanogaster*: 16%, *D. simulans*: 18%, *D. yakuba*: 45%). If the entire complement of genes on the array had been used for normalization, rather than using a set of 1000 genes with sequence conservation, then there would have been an even greater increase in the false positive rate in *D. simulans *(39%) and *D. yakuba *(67%) relative to the platform species, *D. melanogaster *(12%). We also tested this conserved gene normalization strategy with a set of only 100 conserved genes. Normalizing with this reduced set of genes, there is still significant reduction in false positives, but with reduced true positive rate (*D. simulans*: 10% false positives, 29% true positive; *D. yakuba*: 21% false positives, 17% true positive).

### Reduced true positive rate with sequence divergence

**Figure 2:**
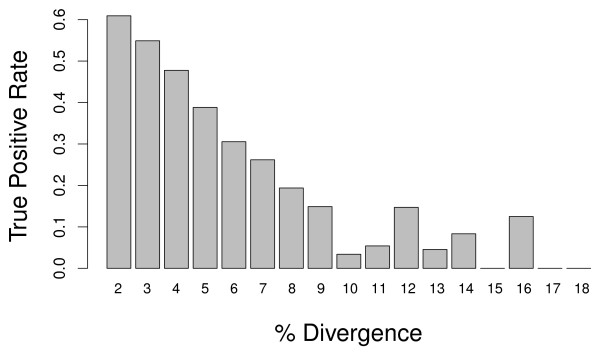
**Success of X detection in *D. simulans*, as influenced by sequence divergence**. Proportion of X features identified as having greater genomic content in *D. simulans *female at each level of sequence divergence from *D. melanogaster*. Features of low sequence divergence (0-1%) are not shown.

In order to determine the extent to which DNA sequence divergence between orthologs hinders the ability to accurately detect increased genome content, we examined the relationship between successful identification of X chromosome orthologs in *D. simulans *and percent identity of the *D. simulans *sequence to that of *D. melanogaster*. We found a strong relationship between percent identity and correct identification. While the true positive rate was roughly 50% for X chromosome *D. simulans *orthologs with a sequence divergence of 2-4%, this rate fell off quickly and bottomed out at about 10% success for orthologs of 9-15% divergence (Figure 2). A similar pattern was observed for *D. yakuba *(data not shown).

### Other genome differences can affect accurate measure of genome content

**Figure 3:**
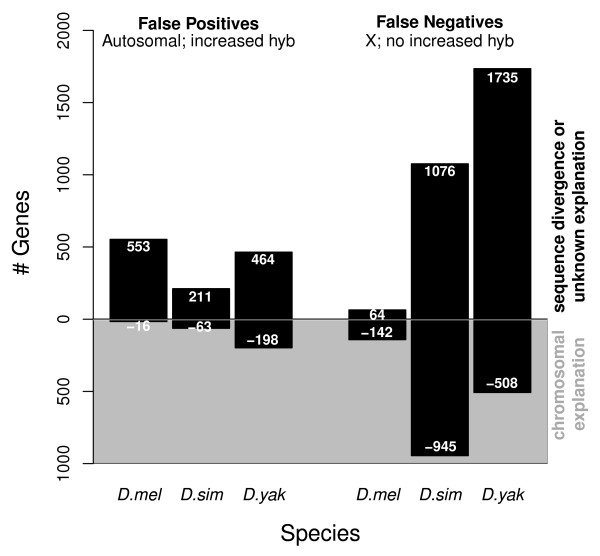
**False positives and negatives explained by sequence divergence, chromosomal factors, or that remain unexplained**.

We accounted for four confounding factors that are expected to decrease the apparent genomic content and thereby lead to false negatives (Table 2). First, we identified *D. melanogaster *X chromosome features that had BLAST hits only to autosomes in the heterologous species (possible deletion or movement off the X). Second, we identified features for which there were a greater number of regions of sequence similarity in *D. melanogaster *than in the heterologous species (differences in copy number or paralogs). Third, we identified features that had no sequence similarity to any region in the heterologous species (possible deletions). Finally, we identified features with hits to heterochromatin in the chromosomal characteristics that might contribute to false negatives. The appropriateness of including this factor is supported by the *D. melanogaster *analysis, where nearly 50% of the features on the array with hits to heterochromatin (130 features total) were either X features not found to be in genomic excess (50) or autosomal features found to be in excess (13). These four confounding factors account for 47% of the false negatives in *D. simulans *and 23% of the false negatives in *D. yakuba *(Figure 3).

**Table 2:** Explanatory chromosomal factors for false negatives.

	False Negatives^a^	No BLAST hit^b^	Autosomal^c^	Tel/het^d^	Mel not Found^e^	Melhit > het^f^	Total Explained^g^
*D. simulans*	2021	228	549	57	185	471	945
*D. yakuba*	2243	231	82	53	168	247	508

^a ^X chromosome features not showing female biased aCGH ratio

^b ^no hit of feature sequence to heterologous genome (E < 10^-14^)

^c ^any hit to an autosome of the heterologous species

^d ^any hit to heterochromatin or telomere region

^e ^not identified as being in excess in *D. melanogaster*

^f ^more hits to *D. melanogaster *genome that to the heterologous genome

^g ^false negatives with one or more chromosomal explanatory factors

We accounted for three confounding factors that may lead to false positives (Table 3). First, we identified *D. melanogaster *autosomal features that had a top BLAST hit to the heterologous X chromosome (possible movement onto the X). Second, we identified *D. melanogaster *autosomal features with a greater number of BLAST hits to the heterologous species than *D. melanogaster *(possible real duplications). We also considered heterochromatic regions as possible false positives. These three confounding factors account for 23% of the false positives in *D. simulans *and 30% of the false positives in *D. yakuba *(Figure 3). Features with hits to telomeric regions were also considered, as these have a greater tendency to duplicate; however, very few incidences of false positive hits to telomeres were found.

**Table 3:** Explanatory chromosomal factors for false positives.

	False Positives^a^	Hit to X^b^	Tel/het^c^	HetHits > mel^d^	Total Explained^e^
*D. simulans*	274	21	6	55	63
*D. yakuba*	662	19	8	186	198

^a ^autosomal features showing female biased aCGH ratio

^b ^top hit to heterologous X chromosome

^c ^any hit to heterochromatin or telomere region

^d ^more hits to heterologous genome that to *D. melanogaster *genome

^e ^false positives with one or more chromosomal explanatory factor

## Discussion

Uncovering incidences and patterns of gene duplication can increase our understanding of this important source of functional novelty [e.g. [43,44]]. It is well documented that aCGH can be used to identify gene dosage, as seen in tumor studies for cancer diagnosis [reviewed by [26]], and in studies of within-species copy number variation [e.g. [23]]. There have also been multiple studies that have successfully used aCGH to identify duplications between species [12,20,39]. Although both hybridization biases resulting from copy number variation for within-species duplicate detection [45,46] and hybridization biases resulting from sequence variation for between-species analysis of single genes [29,40,47] have been addressed, the complexities of duplicate detection under conditions of sequence divergence have not been well addressed. Among the between-species studies of copy number differences in primates, no technical or computational assessment of the influence of sequence divergence has been made. Instead, the result that more lineage-specific copy number increases were found relative to decreases has been taken to indicate that sequence divergence does not significantly contribute to copy number estimates [12]. While full genome sequence does exist for primate species such that a computational validation of aCGH results could be conducted [e.g. [20]], we instead chose an empirical test. We used X-linked array features as a model for duplication and studied three Drosophilid species for which full genome sequence was available. The thousands of X-linked orthologs allowed us to address systematic biases of aCGH duplicate detection that could not have been addressed by the lesser number of known duplicates among Drosophilids [17,48,49]. These systematic biases are introduced by sequence divergence in heterologous the species and by other confounding genomic characteristics related to species divergence.

### Within-species duplicate detection

Consistent with previous aCGH surveys of gene duplication, the 93% true positive rate for *D. melanogaster *X-linked genes demonstrates a strong ability of aCGH to detect copy number variation among individuals of a species. The fact that approximately half of the false negatives had BLAST hits to one or more autosomal sequences reflects an ability to quantify relative genomic content other than straightforward duplication. The significant correlation between the number of similar autosomal sequences and the hybridization ratio reflects the ability to estimate relative copy number. Such a quantitative relationship between copy number and hybridization ratio is integral to cancer diagnostics [50]. Such within-species correlations have been validated repeatedly [26,51]. For example, male and female samples mixed in different amounts were used to assess the ability to identify tumor cells in heterogeneous (tumor and normal) tissues samples [52]. Our within-species results from *D. melanogaster *add evidence that this quantitative relationship persists even when the additional copies do not share perfect sequence identity. As such, the existence of large gene families can interfere with the ability to detect specific gene duplicates with aCGH.

### Duplicate detection in heterologous species will decrease with sequence divergence

Because aCGH relies on sequence similarity for DNA hybridization, sequence dissimilarity of sample DNA to a microarray probe is expected to decrease hybridization of that sample to the array when competitively hybridized with DNA of greater sequence similarity. A roughly linear relationship between sequence divergence and hybridization ratio has been demonstrated repeatedly for single copy genes [29,40,47]. Variation in sequence divergence explains ~ 45-60% of the variation in hybridization ratio [40,53], and our results demonstrate that this will affect our ability to detect gene duplicates in a heterologous species. Successful detection of X-linked genes decreased for heterologous species, and sequence similarity to the array feature had a strong impact on this success. At successively greater sequence divergence there was a lower true positive rate for X chromosome orthologs. When translated to non-model studies of gene duplication among evolutionarily interesting lineages, this predicts of a discovery rate biased toward highly similar gene duplicates.

The biased discovery of highly similar gene duplicates means that many of those recovered by aCGH are likely to be the products of evolutionarily recent events having occurred between closely related species. Therefore, the current results indicate that fewer duplicates will be detected in more distantly related taxa in general, a conclusion that should impact experimental design and phylogenetic inference. Older gene duplicates will be recovered only if they are highly conserved. Such conserved duplicates are thought to be retained when there is a selective advantage for greater protein production of a particular gene product [for review see [1]] as suggested for cold adaptation genes in Antarctic ice fish [10]. Similarly, a selective advantage for spatially or temporally divided expression can produce highly conserved protein coding regions (a type of subfunctionalization) due to mutations in the enhancer regions [54]. Such changes in enhancer regions have been reported to occur in recent duplicates [55]. In some cases, novel function may come about with only a small level of sequence divergence of the protein coding region. Such highly similar duplicates, which can result from a limited number of point mutations, will be retained when the closely related gene products confer a selective advantage, as suggested for the evolution of olfactory receptor family [reviewed by [56]] and opsin genes [e.g. [57]]. Highly conserved duplicates may also be the product of gene conversion [yeast: [58], roundworm: [59]]. Such highly similar duplicates could be recovered with reasonable success by aCGH.

It is important to note that sequence divergence among duplicates is likely to be a complex process, not completely modeled here with the use of the X chromosome. Here we detect a duplication of 1N to 2N and both copies of the gene in the heterologous species exhibit the same percent sequence identity to the array feature. Because competitive hybridization relies on ratios rather than absolute levels, the technique should work equally well for duplications of 2N to 4N, as would occur on autosomes. However, in a natural, between-species comparisons, the gene duplicates present will include those for which two copies are diverged to varying degrees. From the data presented here, it is unclear what the success rate would be for a gene duplicate pair in which one copy was conserved and the other had diverged. This is exactly the case in the proposed processes described by "neofunctionalization" [60]. The rapid evolution of one or both copies following gene duplication has been suggested to accompany adaptive evolution in several instances [e.g. [61,62]]. While theoretical hypotheses regarding the adaptive significance of gene duplicate function or the selective forces that have maintained gene duplicates are tempting, it should be noted that aCGH will also recover gene duplicates that have acquired psuedogene status [63] or that have been fixed in a population due to non-adaptive processes. In all cases, the individual sequence characteristics (GC content, distribution of mismatches, presence of indels, etc.) will influence the hybridization kinetics [40,47,64] and therefore the duplicate discovery rate using aCGH.

### Additional factors affecting duplicate detection

Genomic factors other than sequence divergence can affect duplicate detection in heterologous species. The seven factors that we took into consideration account for a large portion of the false positives and false negatives of the *D. simulans *and *D. yakuba *analyses. If we omit these sets genes from the calculations, our true positive rate for duplicate detection increases to 53% in *D. simulans *and 32% in *D. yakuba*, with the false positive rate reduced to 14% in *D. simulans *and 32% in *D. yakuba*. The remaining false negatives are due to sequence divergence, microarray technical error, or a variable that we did not quantify. However, for gene duplicate discovery in non-model organisms, such detailed sequence information is unlikely to be available and as such would not factor into the analysis. The remaining false positives detected in this study potentially represent actual duplications that were not identified by the BLAST queries due to improper sequence assembly. Algorithms for genome assembly cluster together similar sequences. This legitimately collapses alleles into a single physical location, but also potentially collapses duplicated loci, thus reducing duplications identified by BLAST [19]. However, by determining depth of coverage from raw sequence reads such errors can be addressed and compared to the current array results [e.g. [20]].

### Use of conserved genes for normalization

When detecting duplication levels in heterologous species, it is important to use a normalization method that accounts for hybridization bias [40,41]. Multiple techniques have been proposed for the normalization of aCGH data in order to account for biases due to dramatic sequence divergence in a heterologous test species [65] and the large biases due to extreme copy number, or large segmental duplications associated with cancer [e.g. [45,66]]. In this study, we find support for the use of a set of conserved genes for normalization, such as proposed by van Hijum et al. [65]. In the cross-species experiments, this normalization technique provided a substantial decrease in the false positive rate.

For non-model species lacking substantial genomic DNA sequence data, the set of conserved genes to be used for normalization can be selected according to high sequence conservation across more distantly related and sequenced organisms. Here we use a gene set of 1000 conserved *Drosophila *features for normalization (4.5% of the array). However, we also test a reduced set of only ~100 conserved genes (0.5% of the array features), which represents a gene set size that would be more easily obtainable for species lacking substantial sequence information. This reduced gene set still provides significant reduction in false positives. Van Hijum et al. [65] noted "satisfactory" results using 1.2% array features for normalization, or 20 features per block for print-tip normalization.

## Conclusions

Array CGH can be used to effectively identify gene duplication and genome content; however, certain biases are present due to sequence divergence and other genome characteristics resulting from the divergence between lineages. Using the X chromosome as a proof-of-concept, we demonstrated high true positive rates for genome content in heterologous species. However, we do find a strong negative effect of gene sequence divergence on the ability to identify X-linked genes. X-linked orthologs with less than 90%-95% identity were much less likely to be detected. The false negative rate for these diverged genes should be taken into consideration when making phylogenetic inferences with aCGH because both false positive and false negative rates increase with phylogenetic distance. Furthermore, a biased set of duplicates will be recovered such that those with high sequence similarity will be over-represented. This means that aCGH will be more likely to recover gene duplicates that have been retained due to a selective advantage that relies on conserved gene function, such as increased gene product. Duplicates that have been retained for a selective advantage due to directional selection acting on many loci in one or both copies will be under-represented. Due to this differential representation of gene duplicate classes, one must be cautious when evaluating the relative contribution of different evolutionary processes to the interspecific diversity under study. The aCGH technique is strongly applicable to the growing number of non-model species groups for which a single microarray platform is available. Sequence information provided by EST analysis can be used to select the appropriate set of conserved genes for array normalization that will substantially reduce the false positive rate. Through sequence analysis and functional testing of aCGH-identified gene duplicates, researchers will be able to further address the role of gene duplication in adaptation, speciation, and population dynamics.

## Methods

### Array Production

We used a *Drosophila melanogaster *microarray with ~22,000 features containing PCR products (~500 base pairs long) generated from custom primers designed to predict open reading frames [23] (GEO platform number GPL6056). The microarray was printed on poly-L-lysine slides (Thermo Scientific) in a 48 pin format using an OmniGrid-100 arrayer (GeneMachines). Following hydration, snap drying and UV cross-linking, the slides were blocked with succinic anhydride and sodium borate in 1-Methyl-2-Pyrrolidinone, rinsed, dried according to standard procedure [67] and stored dry until used.

### Sample Preparation and aCGH

Isogenic *Drosophila melanogaster*, *D. simulans *and *D. yakuba *(strain # 14021-0231.36; 14021-0251.195; 14021-0261.01) were obtained from the UCSD *Drosophila *stock center. Genomic DNA from *D. melanogaster *males and virgin females of each species was extracted according to a standard ProteinaseK/Phenol:Chloroform protocol and quantified (Nanodrop 1000). The DNA was diluted to 0.3 μg/μl and size reduced using the Hydroshear (Genome Solutions/Digilab) standard orifice to an average size of 1.5 - 2 Kb, verified by gel electrophoresis. Three micrograms of genomic DNA were fluorescently labeled with Alexa-Fluors conjugated dCTP by Klenow fragment polymerization (Invitrogen, Bio-Prime), the efficiency of which was quantified (Nanodrop 1000) such that competitive hybridizations were matched for DNA concentration. Male *D. melanogaster *samples were used in competitive hybridizations with six female *D. melanogaster *samples, six female *D. simulans *samples, and four female *D. yakuba *samples, in dye swaps to account for dye bias. Hybridizations proceeded for ~16 hours at 65°C in a 3.4× SSC, 0.15% SDS, 1 mM DTT hybridization buffer, or at 48°C in Ambion Hyb Buffer 1, blocked by Cot-1DNA (Invitrogen).

### Microarray Analysis

Hybridized arrays were scanned with an Axon 4000B scanner (Axon Instruments) using Genepix 5.0 software (Axon Instruments). Features of poor quality (fluorescence < 2 standard deviations above background) or known technical error (poor PCR, improper primer design, unexpected PCR length etc.) were flagged and excluded from the analysis. Features that survived this quality control on less than two arrays per species were excluded from the analysis. Raw data from Genepix was imported into R. LIMMA [Linear Models for Microarray Data, [68]] was used to apply a background correction ("minimum") and each of three (see below) within-array intensity normalization ("loess") procedures. Raw and normalized data are submitted to the Gene Expression Omnibus (GEO) repository under the series identifier GSE19584. A linear model was fitted to the data using "lmFit", and "eBayes" provided a correction of variation by borrowing information across genes. Significance was assessed after a FDR multiple testing correction at P < 0.1. In order to not confound statistical power and phylogenetic distance within our study, GEL_50 _measurements of statistical power [69] were held equivalent such that the *D. yakuba *analysis (GEL_50 _= 0.456) employed 4 hybridizations and the *D. simulans *analysis (GEL_50 _= 0.455) employed 6 hybridizations. This difference in required technical replication was likely due to array quality.

### Assessment of Normalization

Since we expect the normalization of cross-species arrays to be affected by a substantial number of diverged genes in the non-platform species (and in this case, also a substantial number of duplicated loci), we compared our preferred normalization technique, using a set of ~1000 genes that are highly conserved among the three *Drosophila *species (determined with NCBI megaBLAST 1/2009), to a normalization based on a set of 100 conserved genes and to a traditional normalization using the full complement of array features. In each case, statistical analysis for hybridization bias was performed (see above).

### Sequence Analysis

Features representing X chromosome genes were identified by the top BLAST hit of the feature sequence to *D. melanogaster *genome sequence. Sequence divergence of X chromosome orthologs was assessed as the percent sequence identity (%ID) of the feature sequence to the top BLAST hit in *D. simulans *and *D. yakuba *genome sequence (E < 10^-14^, NCBI megaBLAST 1/2009).

Additional autosomal regions of high sequence similarity in each species were identified (E < 10^-14^) and a measure of the relative number of occurrences of a sequence in the two genomes of interest was determined as the ratio of the number of BLAST hits to the heterologous species to number of BLAST hits to the reference species (E < 10^-10^). Movement off of the X chromosome was predicted by the lack of any BLAST hit of the feature sequence to the heterologous X chromosome. Conversely movement onto the X chromosome was predicted by the presence of any BLAST hit of an autosomal feature sequence to the heterologous X chromosome. Furthermore, any hits to heterochromatin or to telomeric regions (25 Kb from the end of a chromosome) were also recorded. These factors represent between-genome chromosomal differences that can confound duplicate detection for cross-species aCGH analyses.

## Authors' contributions

SCPR and HEM performed microarray hybridizations. HEM performed the statistical analysis. SCPR conceived of the project. Both SCPR and HEM wrote and have read and approved the manuscript.

## References

[B1] Taylor JS Raes J Duplication and divergence: The evolution of new genes and old ideas Annu Rev Genet 2004 38 615 643 10.1146/annurev.genet.38.072902.092831 15568988

[B2] Zhang JZ Rosenberg HF Nei M Positive Darwinian selection after gene duplication in primate ribonuclease genes Proc Natl Acad Sci USA 1998 95 7 3708 3713 10.1073/pnas.95.7.3708 9520431 PMC19901

[B3] Zhang JZ Zhang YP Rosenberg HF Adaptive evolution of a duplicated pancreatic ribonuclease gene in a leaf-eating monkey Nat Genet 2002 30 4 411 415 10.1038/ng852 11925567

[B4] Perry GH Dominy NJ Claw KG Lee AS Fiegler H Redon R Werner J Villanea FA Mountain JL Misra R Diet and the evolution of human amylase gene copy number variation Nat Genet 2007 39 10 1256 1260 10.1038/ng2123 17828263 PMC2377015

[B5] Labbe P Berthomieu A Berticat C Alout H Raymond M Lenormand T Weill M Independent duplications of the acetylcholinesterase gene conferring insecticide resistance in the mosquito *Culex pipiens* Mol Biol Evol 2007 24 4 1056 1067 10.1093/molbev/msm025 17283366

[B6] Daborn PJ Yen JL Bogwitz MR Le Goff G Feil E Jeffers S Tijet N Perry T Heckel D Batterham P A single P450 allele associated with insecticide resistance in *Drosophila* Science 2002 297 5590 2253 2256 10.1126/science.1074170 12351787

[B7] Ting CT Tsaur SC Sun S Browne WE Chen YC Patel NH Wu CL Gene duplication and speciation in *Drosophila*: Evidence from the Odysseus locus Proc Natl Acad Sci USA 2004 101 33 12232 12235 10.1073/pnas.0401975101 15304653 PMC514461

[B8] Lynch M Force AG The origin of interspecific genomic incompatibility via gene duplication Am Nat 2000 156 6 590 605 10.1086/316992 29592543

[B9] Sandve SR Rudi H Asp T Rognli OA Tracking the evolution of a cold stress associated gene family in cold tolerant grasses BMC Evol Biol 2008 8 10.1186/1471-2148-8-245 18775065 PMC2542378

[B10] Chen ZZ Cheng CHC Zhang JF Cao LX Chen L Zhou LH Jin YD Ye H Deng C Dai ZH Transcrintomic and genomic evolution under constant cold in Antarctic notothenioid fish Proc Natl Acad Sci USA 2008 105 35 12944 12949 10.1073/pnas.0802432105 18753634 PMC2529033

[B11] Monson RK Gene duplication, neofunctionalization, and the evolution of C-4 photosynthesis International Journal of Plant Sciences 2003 164 3 S43 S54 10.1086/368400

[B12] Dumas L Kim YH Karimpour-Fard A Cox M Hopkins J Pollack JR Sikela JM Gene copy number variation spanning 60 million years of human and primate evolution Genome Res 2007 17 9 1266 1277 10.1101/gr.6557307 17666543 PMC1950895

[B13] Bailey JA Gu ZP Clark RA Reinert K Samonte RV Schwartz S Adams MD Myers EW Li PW Eichler EE Recent segmental duplications in the human genome Science 2002 297 5583 1003 1007 10.1126/science.1072047 12169732

[B14] Bailey JA Church DM Ventura M Rocchi M Eichler EE Analysis of segmental duplications and genome assembly in the mouse Genome Res 2004 14 5 789 801 10.1101/gr.2238404 15123579 PMC479105

[B15] Feuk L Carson AR Scherer SW Structural variation in the human genome Nature Reviews Genetics 2006 7 2 85 97 10.1038/nrg1767 16418744

[B16] Hughes T Liberles DA The pattern of evolution of smaller-scale gene duplicates in mammalian genomes is more consistent with neo-than subfunctionalisation J Mol Evol 2007 65 5 574 588 10.1007/s00239-007-9041-9 17957399

[B17] Hahn MW Han MV Han SG Gene family evolution across 12 *Drosophila *genomes PLoS Genet 2007 3 11 2135 2146 10.1371/journal.pgen.0030197 PMC2065885 17997610

[B18] Moore RC Purugganan MD The evolutionary dynamics of plant duplicate genes Curr Opin Plant Biol 2005 8 2 122 128 10.1016/j.pbi.2004.12.001 15752990

[B19] Eichler EE Segmental duplications: What's missing, misassigned, and misassembled - and should we care? Genome Res 2001 11 5 653 656 10.1101/gr.188901 11337463

[B20] Marques-Bonet T Kidd JM Ventura M Graves TA Cheng Z Hillier LW Jiang ZS Baker C Malfavon-Borja R Fulton LA A burst of segmental duplications in the genome of the African great ape ancestor Nature 2009 457 7231 877 881 10.1038/nature07744 19212409 PMC2751663

[B21] Yoon ST Xuan ZY Makarov V Ye K Sebat J Sensitive and accurate detection of copy number variants using read depth of coverage Genome Res 2009 19 9 1586 1592 10.1101/gr.092981.109 19657104 PMC2752127

[B22] Redon R Ishikawa S Fitch KR Feuk L Perry GH Andrews TD Fiegler H Shapero MH Carson AR Chen WW Global variation in copy number in the human genome Nature 2006 444 7118 444 454 10.1038/nature05329 17122850 PMC2669898

[B23] Dopman EB Hartl DL A portrait of copy-number polymorphism in *Drosophila melanogaster* Proc Natl Acad Sci USA 2007 104 50 19920 19925 10.1073/pnas.0709888104 18056801 PMC2148398

[B24] Bloomfield G Tanaka Y Skelton J Ivens A Kay RR Widespread duplications in the genomes of laboratory stocks of *Dictyostelium discoideum* Genome Biol 2008 9 4 10.1186/gb-2008-9-4-r75 18430225 PMC2643946

[B25] Lynch M Sung W Morris K Coffey N Landry CR Dopman EB Dickinson WJ Okamoto K Kulkarni S Hartl DL A genome-wide view of the spectrum of spontaneous mutations in yeast Proc Natl Acad Sci USA 2008 105 27 9272 9277 10.1073/pnas.0803466105 18583475 PMC2453693

[B26] Pinkel D Albertson DG Comparative genomic hybridization Annu Rev Genomics Hum Genet 2005 6 331 354 10.1146/annurev.genom.6.080604.162140 16124865

[B27] West MAL van Leeuwen H Kozik A Kliebenstein DJ Doerge RW St Clair DA Michelmore RW High-density haplotyping with microarray-based expression and single feature polymorphism markers in *Arabidopsis* Genome Res 2006 16 6 787 795 10.1101/gr.5011206 16702412 PMC1473188

[B28] Miller MR Dunham JP Amores A Cresko WA Johnson EA Rapid and cost-effective polymorphism identification and genotyping using restriction site associated DNA (RAD) markers Genome Res 2007 17 2 240 248 10.1101/gr.5681207 17189378 PMC1781356

[B29] Brunelle BW Nicholson TL Stephens RS Microarray-based genomic surveying of gene polymorphisms in *Chlamydia trachomatis* Genome Biol 2004 5 6 9 10.1186/gb-2004-5-6-r42 PMC463075 15186493

[B30] Giuntini E Mengoni A De Filippo C Cavalieri D Aubin-Horth N Landry CR Becker A Bazzicalupo M Large-scale genetic variation of the symbiosis-required megaplasmid pSymA revealed by comparative genomic analysis of *Sinorhizobium meliloti *natural strains BMC Genomics 2005 6 10.1186/1471-2164-6-158 16283928 PMC1298293

[B31] Janvilisri T Scaria J Thompson AD Nicholson A Limbago BM Arroyo LG Songer JG Grohn YT Chang YF Microarray Identification of *Clostridium difficile *Core Components and Divergent Regions Associated with Host Origin J Bacteriol 2009 191 12 3881 3891 10.1128/JB.00222-09 19376880 PMC2698405

[B32] Zhou DS Han YP Dai EH Pei DC Song YJ Zhai JH Du ZM Wang J Guo ZB Yang RF Identification of signature genes for rapid and specific characterization of *Yersinia pestis* Microbiol Immunol 2004 48 4 263 269 15107536 10.1111/j.1348-0421.2004.tb03522.x

[B33] Hinchliffe SJ Isherwood KE Stabler RA Prentice MB Rakin A Nichols RA Oyston PCF Hinds J Titball RW Wren BW Application of DNA mcroarrays to study the evolutionary genomics of *Yersinia pestis *and *Yersinia pseudotuberculosis* Genome Res 2003 13 9 2018 2029 10.1101/gr.1507303 12952873 PMC403674

[B34] Kato-Maeda M Rhee JT Gingeras TR Salamon H Drenkow J Smittipat N Small PM Comparing genomes within the species *Mycobacterium tuberculosis *(vol 11, pg 547, 2001) Genome Res 2001 11 10 1796 1796 10.1101/gr166401 PMC311074 11282970

[B35] Dziejman M Balon E Boyd D Fraser CM Heidelberg JF Mekalanos JJ Comparative genomic analysis of *Vibrio cholerae*: Genes that correlate with cholera endemic and pandemic disease Proc Natl Acad Sci USA 2002 99 3 1556 1561 10.1073/pnas.042667999 11818571 PMC122229

[B36] Turner TL Hahn MW Nuzhdin SV Genomic islands of speciation in *Anopheles gambiae* PLoS Biol 2005 3 9 1572 1578 10.1371/journal.pbio.0030285 PMC1182689 16076241

[B37] Riehle MM Markianos K Niare O Xu JN Li J Toure AM Podiougou B Oduol F Diawara S Diallo M Natural malaria infection in *Anopheles gambiae *is regulated by a single genomic control region Science 2006 312 5773 577 579 10.1126/science.1124153 16645095

[B38] Locke DP Segraves R Carbone L Archidiacono N Albertson DG Pinkel D Eichler EE Large-scale variation among human and great ape genomes determined by array comparative genomic hybridization Genome Res 2003 13 3 347 357 10.1101/gr.1003303 12618365 PMC430292

[B39] Fortna A Kim Y MacLaren E Marshall K Hahn G Meltesen L Brenton M Hink R Burgers S Hernandez-Boussard T Lineage-specific gene duplication and loss in human and great ape evolution PLoS Biol 2004 2 7 937 954 10.1371/journal.pbio.0020207 PMC449870 15252450

[B40] Renn SCP Machado HE Jones A Soneji K Kulathinal RJ Hofmann HA Using comparative genomic hybridization to survey genomic sequence divergence across species: A proof-of-concept from *Drosophila* BMC Genomics 2010 11 271 10.1186/1471-2164-11-271 20429934 PMC2873954

[B41] Murray AE Lies D Li G Nealson K Zhou J Tiedje JM DNA/DNA hybridization to microarrays reveals gene-specific differences between closely related microbial genomes Proc Natl Acad Sci USA 2001 98 17 9853 9858 10.1073/pnas.171178898 11493693 PMC55542

[B42] Clark AG Eisen MB Smith DR Bergman CM Oliver B Markow TA Kaufman TC Kellis M Gelbart W Iyer VN Evolution of genes and genomes on the *Drosophila *phylogeny Nature 2007 450 7167 203 218 10.1038/nature06341 17994087

[B43] Blomme T Vandepoele K De Bodt S Simillion C Maere S Peer Y Van de The gain and loss of genes during 600 million years of vertebrate evolution Genome Biol 2006 7 5 10.1186/gb-2006-7-5-r43 16723033 PMC1779523

[B44] Li L Huang YW Xia XF Sun ZR Preferential duplication in the sparse part of yeast protein interaction network Mol Biol Evol 2006 23 12 2467 2473 10.1093/molbev/msl121 16980576

[B45] Khojasteh M Lam WL Ward RK MacAulay C A stepwise framework for the normalization of array CGH data BMC Bioinformatics 2005 6 10.1186/1471-2105-6-274 16297240 PMC1310623

[B46] Staaf J Jonsson G Ringner M Vallon-Christersson J Normalization of array-CGH data: influence of copy number imbalances BMC Genomics 2007 8 10.1186/1471-2164-8-382 PMC2190775 17953745

[B47] Taboada EN Acedillo RR Luebbert CC Findlay WA Nash JHE A new approach for the analysis of bacterial microarray-based Comparative Genomic Hybridization: insights from an empirical study BMC Genomics 2005 6 1 10 10.1186/1471-2164-6-78 15918914 PMC1168892

[B48] Thornton K Long M Rapid divergence of gene duplicates on the *Drosophila melanogaster *X chromosome Mol Biol Evol 2002 19 6 918 925 12032248 10.1093/oxfordjournals.molbev.a004149

[B49] Osada N Innan H Duplication and Gene Conversion in the *Drosophila melanogaster *Genome PLoS Genet 2008 4 12 10.1371/journal.pgen.1000305 19079581 PMC2588116

[B50] Pollack JR Sorlie T Perou CM Rees CA Jeffrey SS Lonning PE Tibshirani R Botstein D Borresen-Dale AL Brown PO Microarray analysis reveals a major direct role of DNA copy number alteration in the transcriptional program of human breast tumors Proc Natl Acad Sci USA 2002 99 20 12963 12968 10.1073/pnas.162471999 12297621 PMC130569

[B51] Pinkel D Seagraves R Sudar D Clark S Poole I Kowbel D Collins C Kuo WL Chen C Zhai Y High resolution analysis of DNA copy number variation using comparative genomic hybridization to microarrays Nat Genet 1998 20 2 207 211 10.1038/2524 9771718

[B52] Garnis C Coe BP Lam SL MacAulay C Lam WL High-resolution array CGH increases heterogeneity tolerance in the analysis of clinical samples Genomics 2005 85 6 790 793 10.1016/j.ygeno.2005.02.015 15885505

[B53] Dong YM Glasner JD Blattner FR Triplett EW Genomic interspecies microarray hybridization: Rapid discovery of three thousand genes in the maize endophyte, *Klebsiella pneumoniae *342, by microarray hybridization with *Escherichia coli *K-12 open reading frames Appl Environ Microbiol 2001 67 4 1911 1921 10.1128/AEM.67.4.1911-1921.2001 11282649 PMC92813

[B54] Huminiecki L Wolfe KH Divergence of spatial gene expression profiles following species-specific gene duplications in human and mouse Genome Res 2004 14 10A 1870 1879 10.1101/gr.2705204 15466287 PMC524410

[B55] Kohn MH Rapid sequence divergence rates in the 5 prime regulatory regions of young *Drosophila melanogaster *duplicate gene pairs Genetics and Molecular Biology 2008 31 2 575 584 10.1590/S1415-47572008000300028

[B56] Kratz E Dugas JC Ngai J Odorant receptor gene regulation: implications from genomic organization Trends Genet 2002 18 1 29 34 10.1016/S0168-9525(01)02579-3 11750698

[B57] Smith AR Carleton KL Intrageneric Sequence Diversity in Cichlid Opsin Arrays Integrative and Comparative Biology 2009 49 E307 E307

[B58] Gangloff S Zou H Rothstein R Gene conversion plays the major role in controlling the stability of large tandem repeats in yeast EMBO J 1996 15 7 1715 1725 8612596 PMC450084

[B59] Semple C Wolfe KH Gene duplication and gene conversion in the *Caenorhabditis elegans *genome J Mol Evol 1999 48 5 555 564 10.1007/PL00006498 10198121

[B60] Force A Lynch M Pickett FB Amores A Yan YL Postlethwait J Preservation of duplicate genes by complementary, degenerative mutations Genetics 1999 151 4 1531 1545 10101175 10.1093/genetics/151.4.1531 PMC1460548

[B61] Han MV Demuth JP McGrath CL Casola C Hahn MW Adaptive evolution of young gene duplicates in mammals Genome Res 2009 19 5 859 867 10.1101/gr.085951.108 19411603 PMC2675974

[B62] Adams KL Wendel JF Polyploidy and genome evolution in plants Curr Opin Plant Biol 2005 8 2 135 141 10.1016/j.pbi.2005.01.001 15752992

[B63] Lynch M Conery JS The evolutionary fate and consequences of duplicate genes Science 2000 290 5494 1151 1155 10.1126/science.290.5494.1151 11073452

[B64] Bar-Or C Czosnek H Koltai H Cross-species microarray hybridizations: a developing tool for studying species diversity Trends Genet 2007 23 4 200 207 10.1016/j.tig.2007.02.003 17313995

[B65] van Hijum S Baerends RJS Zomer AL Karsens HA Martin-Requena V Trelles O Kok J Kuipers OP Supervised Lowess normalization of comparative genome hybridization data - application to lactococcal strain comparisons BMC Bioinformatics 2008 9 10.1186/1471-2105-9-93 PMC2275246 18267014

[B66] van Houte BPP Binsl TW Hettling H Pirovano W Heringa J CGHnormaliter: an iterative strategy to enhance normalization of array CGH data with imbalanced aberrations BMC Genomics 2009 10 10.1186/1471-2164-10-401 PMC2748095 19709427

[B67] Hegde P Qi R Abernathy K Gay C Dharap S Gaspard R Hughes JE Snesrud E Lee N Quackenbush J A concise guide to cDNA microarray analysis Biotechniques 2000 29 3 548 10997270 10.2144/00293bi01

[B68] Smyth GK Linear models and empirical Bayes methods for assessing differential expression in microarray experiments Stat Appl Genet Mol Biol 2004 3 1 26 10.2202/1544-6115.1027 16646809

[B69] Townsend JP Resolution of large and small differences in gene expression using models for the Bayesian analysis of gene expression levels and spotted DNA microarrays BMC Bioinformatics 2004 5 13 10.1186/1471-2105-5-54 15128431 PMC420235

